# Abscopal Effects of Local Radiotherapy Are Dependent on Tumor Immunogenicity

**DOI:** 10.3389/fonc.2021.690188

**Published:** 2021-06-24

**Authors:** Jin-Zhi Lai, Yan-Yang Zhu, Ying Liu, Lin-Lin Zhou, Li Hu, Ling Chen, Qiu-Yu Zhang

**Affiliations:** ^1^ Institute of Immunotherapy, Fujian Medical University, Fuzhou, China; ^2^ Department of Oncology, The Second Affiliated Hospital of Fujian Medical University, Quanzhou, China

**Keywords:** radiation, abscopal effect, immunogenicity, tumor microenvironment, CD8 T cells

## Abstract

Although abscopal tumor regression remains a rare phenomenon, interest in exploiting how radiation stimulates the immune system to induce systemic abscopal response is increasing. Here, we tested the hypothesis that tumor immunogenicity determined the ability of radiotherapy to induce abscopal effects. We established highly (MC-38 and E.G7-OVA) or poorly (LL/2 and B16-F10) immunogenic tumor models in this study and treated them with sham radiation, a single dose of 15 Gy, or three fractions of 5 Gy on three consecutive days. Alterations in the tumor microenvironment after radiation were examined by flow cytometry and RNA sequencing. Our results demonstrated the positive correlation between tumor immunogenicity and the abscopal effect of radiotherapy. The single dose of 15 Gy radiation was an effective regimen for inducing abscopal effects in highly immunogenic tumors. Local radiation reshaped the tumor microenvironment of irradiated and non-irradiated distant tumors by increasing CD8 T-cell infiltration and reducing suppressive immune cell accumulation. However, radiation alone was insufficient to elicit abscopal effects in poorly immunogenic tumors. No significant alterations were detected in the non-irradiated distant tumor microenvironment after radiation of poorly immunogenic tumors. In addition, tumor immunogenic subtypes were associated with the radiological response and clinical outcome of patients receiving radiotherapy. These findings indicated that tumor immunogenicity was the dominant characteristic that could predict the abscopal effect of radiotherapy. Our study provides an in-depth understanding of the immunological mechanisms involved in abscopal effects and highlights the impact of tumor heterogeneity on the therapeutic efficacy of radiotherapy and their combination with immunotherapy in clinical trials.

## Introduction

Radiotherapy is the standard-of-care treatment for localised cancers and palliative treatment in metastatic disease (1). The abscopal effect is a phenomenon in which local radiotherapy is associated with the regression of metastatic cancer outside of the irradiated field (2). Currently, most researchers believe that radiation induces oxidative stress or injury in tumors, thus leading to the liberation of neoantigens and cellular damage-associated molecular patterns (DAMPs), such as tumor-associated antigens, necrotic tumor cells and debris. A significant increase in the diversity and number of neoantigens can activate a tumor-specific immune response, with tumor-associated antigens recognised by antigen presenting cells (APCs) and then presented to effector T cells. Effector T cells can then recognise and eliminate both irradiated tumors and metastatic tumors (3). Although the abscopal effect is a rare event in metastatic tumor patients receiving radiotherapy alone, the growing consensus is that radiotherapy combined with immunotherapy provides an opportunity to boost the abscopal effect in some clinical trials (4–6). To date, the biological mechanisms underlying the abscopal effect in different tumor types are not yet fully understood.

The goals of our study were to explore the cellular and molecular mechanisms by which radiotherapy reshaped the tumor microenvironment and induced abscopal effects in murine models. Our study strongly indicated that the radiation-induced abscopal effect was positively correlated with tumor immunogenicity, which is the ability of cancer cells to induce adaptive immune responses (7). Cancer cells that can elicit a protective immune response to inhibit tumor growth are considered to have high immunogenicity. Conversely, cancer cells that only stimulate a weak immune response and fail to control tumor growth are classified as having poor immunogenicity (8). In this study, we observed the efficacy of radiotherapy in both highly and poorly immunogenic tumor models and found that single high-dose radiation was optimal for stimulating a localised antitumor immune response and provided an opportunity to boost abscopal response rates in highly immunogenic tumors. Nevertheless, radiation alone was insufficient to elicit abscopal effects in poorly immunogenic tumors. Therefore, the abscopal effect of radiation appears to be correlated with tumor immunogenicity.

## Materials and Methods

### Mouse Strains and Cell Lines

Female C57BL/6 mice (age, 6–8 weeks) were purchased from Beijing Vital River (Beijing, China) and housed in pathogen-free facilities in the Experimental Animal Centre of Fujian Medical University.

Most mouse tumor cell lines were purchased from ATCC (Manassas, USA), including B16-F10 melanoma cells, LL/2 Lewis lung carcinoma cells (LLC1) and E.G7-OVA OVA-expressing EL4 thymic lymphoma cells. MC38 colorectal carcinoma cells were obtained from the laboratory of Dr. Lieping Chen (Yale University). All tumor cell lines were tested to be free of mycoplasma before use.

### Animal Experiments

A total of 0.5 × 10^6^ MC-38, E.G7-OVA, LL/2 or B16-F10 cells were injected subcutaneously into the right flank (irradiated tumors) and left flank (non-irradiated tumors) of C57BL/6 mice. The perpendicular tumor diameters were calculated using the equation (l + w)/2, where l and w refer to the larger and smaller dimensions, respectively. Mice were randomised into different treatment groups when the irradiated tumor diameters reached 5–6 mm. In the irradiation group, mice were anaesthetised with isoflurane and placed under lead shielding with a 15 mm diameter aperture aligned over the tumor. Only the irradiated tumor in the right flank was exposed to irradiation while the rest of the body was kept outside the radiation field. Radiation was performed using an RS-2000 Biological Irradiator (RadSource, Canada) at 160 kV, 10 mA and a dose rate of 1.05 Gy/min. Depletion of CD8 T cells was achieved by intraperitoneal injection of 200 µg of CD8-depleting antibody (anti-mouse CD8α, clone 53.6.7) once a week three consecutive times. All the mice were regarded as dead from humane treatment after the irradiated or non-irradiated tumors reached 20 mm in size for each dimension.

### Flow Cytometry

Single-cell populations were isolated from fresh tumor tissue using a Gentle MACS mechanical dissociator in the presence of lysis buffers (Miltenyi Biotec, Germany). Cells were blocked with anti-mouse CD16/32 (TruStain fcX, USA) and then stained with antibodies against mouse CD3e, CD4, CD8a, CD45.2, CD11b, F4/80, Gr-1, CD25, PD-1 (programmed cell death protein-1), TIM-3 (T cell immunoglobulin domain and mucin domain-3), Foxp3 (forkhead box P3), IFN-γ, TNF-α, death marker and matched isotype controls depending on the experiment. For cytokine staining, the cells were restimulated with ionomycin and PMA (phorbol 12-myristate 13-acetate) for 4 h in the presence of GolgiPlug (BD Biosciences, USA) before intracellular staining. The gating strategy is shown in **Supplementary Figure 1A**. These antibodies and staining agents were obtained from Thermo Scientific and BD Biosciences. Samples were run on a BD FACSVerse system and analysed using FlowJo software version 10 (BD Biosciences, USA).

### RNA Isolation and Sequencing

RNA sequencing was performed on samples isolated 8 days after the first dose of radiation. Total RNA from the irradiated tumors, non-irradiated tumors and sham-irradiated tumors was extracted using TRIzol (Sangon, China) according to the manufacturer’s instructions. To construct Illumina sequencing libraries, a total amount of 2 μg RNA per sample was used as the input material for the RNA sample preparations. Sequencing libraries were generated using the VAHTSTM mRNA-seq V2 Library Prep Kit for Illumina^®^ following the manufacturer’s recommendations, and index codes were added to attribute sequences to each sample. The libraries were subsequently quantified and pooled. Paired-end sequencing of the libraries was performed on HiSeq XTen sequencers (Illumina, USA). Normalised and log2-transformed TPM values from RNA-Seq based on the expectation maximisation data, which reflect relative mRNA expression, were analysed using the Mann–Whitney test.

### Immune Cell Analysis and Gene Functional Enrichment Analysis

The Immune Cell Abundance Identifier (ImmuCellAI-mouse, http://bioinfo.life.hust.edu.cn/ImmuCellAI-mouse/#!/) has been recently developed to estimate the abundance of 36 immune cell types, including 11 T-cell subsets, from mouse gene expression data (9). For each queried sample, the enrichment score of the total expression deviation of the signal gene sets was calculated and assigned to each immune cell type by the Single-sample Gene Set Enrichment Analysis (ssGSEA) algorithm. GSEA (https://software.broadinstitute.org/gsea/index.jsp) was applied to analyse signalling pathway enrichment in non-irradiated and control tumors using the KEGG database in MSigDB (version 7.3). The enriched pathways were arranged in the order of their normalised enrichment scores (NESs), and *p <*0.05 and FDR <0.25 were considered statistically significant in the GSEA analyses.

### Bioinformatics Data Analysis

The following three independent datasets were downloaded from the Gene Expression Omnibus (GEO) database: GSE35452 (rectal cancer), GSE116918 (prostate cancer), and GSE7696 (glioblastoma). We first quantified the enrichment levels of the 29 immune signatures in each sample by the single-sample gene-set enrichment analysis (ssGSEA) score (10). Next, the ssGSEA scores for each immune cell type were standardised and tumors were divided into a predominant immune group (PI) and a low immune group (LI) using Ward’s hierarchical clustering method. Kaplan–Meier survival curve analysis was used to analyse survival between the PI and LI groups.

### Statistical Analysis

Statistical analyses were performed using Prism 8 (GraphPad, Canada). All data are shown as the mean ± SD unless otherwise stated, and significant differences were determined using a two-tailed Student’s t-test or one-way ANOVA with Tukey’s multiple comparison test. The survival difference was analysed by Wilcoxon and log-rank tests. *p <*0.05 was considered statistically significant. The results represent at least two experiments unless otherwise stated.

## Results

### Tumors Were Stratified as Highly or Poorly Immunogenic According to the Number of Infiltrating Immune Cells and Expression of MHC-I Molecules

**Figure 1:**
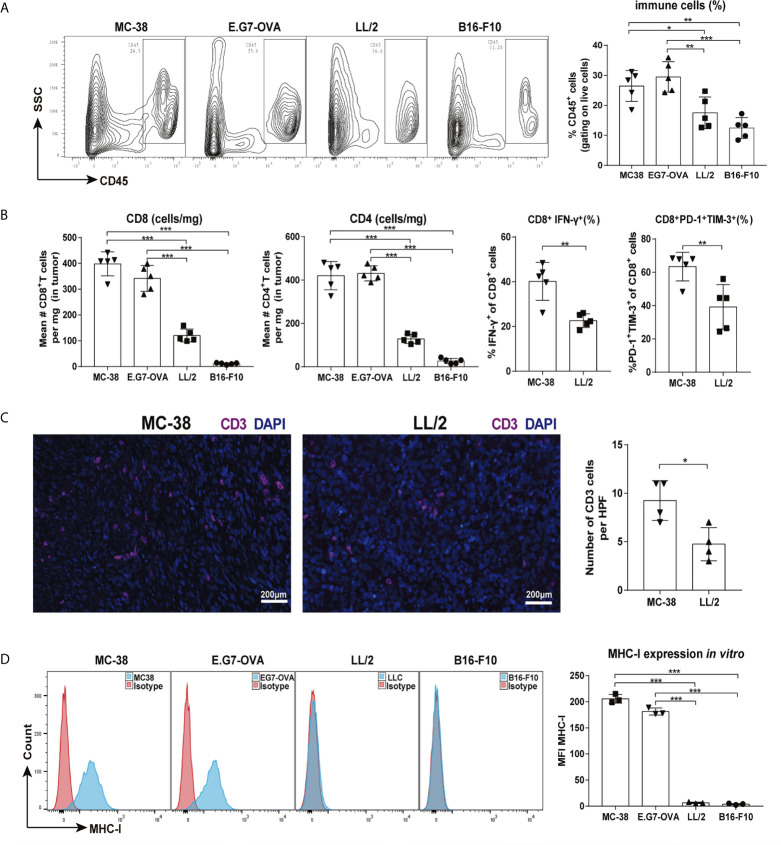
Tumor immunogenic levels of four murine tumors. Mice were inoculated with MC-38, E.G7-OVA, LL/2 and B16-F10 tumors, and then tumor infiltrating cells were isolated for cytometry analysis when the tumor lengths reached approximately 5–6 mm (on days 7–9). **(A)** Percentages of CD45-positive lymphocytes in four types of tumor tissues are shown in representative FACS plots (left graph) and pooled from two independent experiments. **(B)** Absolute numbers of CD8 T and CD4 T cells in four types of tumor tissues were counted, and percentages of CD8^+^IFN-γ^+^ and CD8^+^PD-1^+^TIM-3^+^ cells were analysed in MC-38 and LL/2 tumor tissues. **(C)** Infiltrating T cells in MC-38 and LL/2 tumors were stained with anti-CD3 (purple) and DAPI (blue, nuclei staining) and detected by immunofluorescence. Scale bars, 200 μm. **(D)** Histograms of MHC-I molecule expression on MC-38, E.G7-OVA, LL/2 and B16-F10 cells *in vitro*. Representative results from one of at least three independent experiments are shown. *p < 0.05, **p < 0.01, ***p < 0.001.

Tumor immunogenicity is defined as the ability of a tumor to stimulate an immune response that can inhibit tumor growth (7). Here, we assessed the immunogenicity of four murine tumor models (MC-38, E.G7-OVA, LL/2, and B16-F10). The percentages of tumor-infiltrating immune cells (gating on live cells) were higher in the MC-38 and E.G7-OVA tumors than the LL/2 and B16-F10 tumors (**Figure 1A**). The absolute numbers of CD8 and CD4 T cells were significantly increased in the MC-38 and E.G7-OVA tumor tissues. In addition, CD8 T cells displayed higher levels of PD-1, TIM-3 and IFN-γ in the MC-38 tumors than the LL/2 tumors (**Figure 1B** and **Supplementary Figure 1B**). Higher infiltration of CD3 T cells was also observed in the MC-38 tumors than the LL/2 tumors by immunofluorescence staining (**Figure 1C**). Previous studies have identified a significant correlation between MHC-I expression in tumor cells and immunogenicity (11). Our data showed that MHC-I (H-2Kb) expression was high in the MC-38 and E.G7-OVA cells but not observed in the LL/2 and B16-F10 cells (**Figure 1D**). In addition, the RNA-seq analysis revealed that the expression of multiple immune-related genes was upregulated in the MC-38 tumors but not the LL/2 tumors (**Supplementary Figure 1C**). Altogether, our results indicated that MC-38 and E.G7-OVA cells were highly immunogenic tumor cells while LL/2 and B16-F10 cells were poorly immunogenic tumor cells.

### Radiation-Induced Abscopal Effect Was Associated With Tumor Immunogenicity and the Radiotherapy Regimen

**Figure 2:**
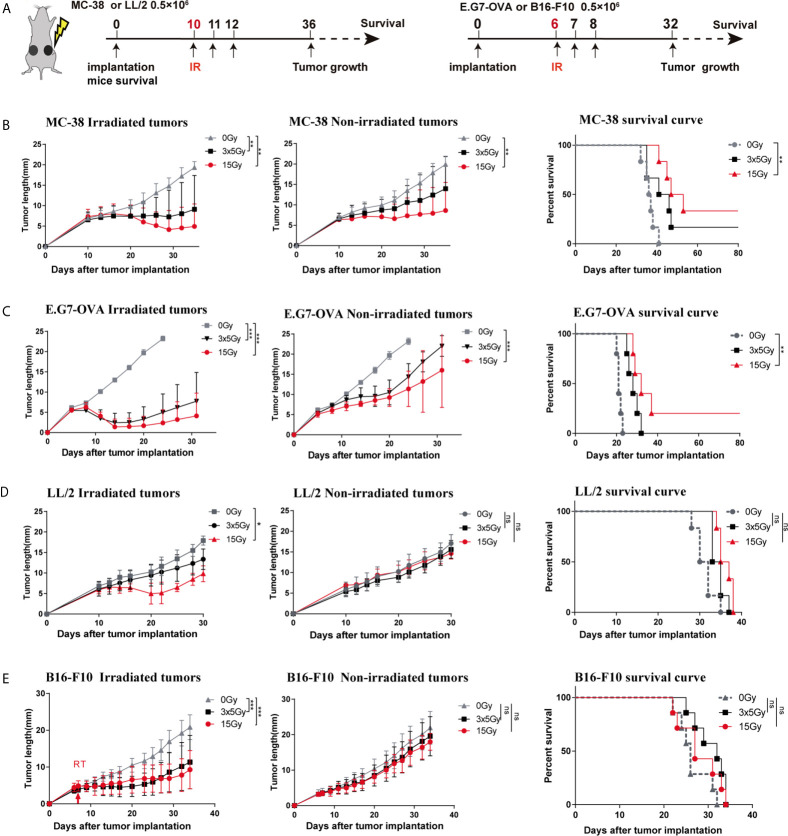
Response to radiotherapy in highly and poorly immunogenic tumors. **(A)** Four types of murine tumor cells were subcutaneously inoculated in the bilateral flanks of C57BL/6 mice. Irradiated (in the right flank) tumors received 3 × 5 Gy, 15 Gy or sham radiation when tumor lengths reached 5–6 mm. **(B–E)** Left: Tumor growth curve of irradiated and non-irradiated tumors treated with 3 × 5 Gy, 15 Gy or sham radiation in the **(B)** MC-38 model (n = 12), **(C)** E.G7-OVA model (n = 10), **(D)** LL/2 model (n = 12) and **(E)** B16-F10 model (n = 12); Right: Survival rate of the mice in each group for the MC-38, E.G7-OVA, LL/2 and B16-F10 models. Percent survival of mice in the different groups depicted with a Kaplan–Meier plot. One-way ANOVA was used to compare tumor sizes at the endpoint of these three groups *p < 0.05, **p < 0.01, ***p < 0.001, ns, no significance.

Highly immunogenic tumors (MC-38 and E.G7-OVA) and poorly immunogenic tumors (LL/2 and B16-F10) were used to establish bilateral tumor models (**Figure 2A**). We observed that radiotherapy (15 Gy or 3 × 5 Gy) significantly inhibited irradiated tumor growth and induced abscopal effects in both the MC-38 and E.G7-OVA tumor models (**Figures 2B, C**, left and middle panels). Furthermore, the percentages of both irradiated and non-irradiated tumors with complete regression were higher with a single dose of 15 Gy radiation than with three doses of 5 Gy radiation (**Supplementary Figures 2A, B**). Moreover, we also found that 15 Gy radiation prolonged mouse survival in the MC-38 and E.G7-OVA models (**Figures 2B, C**, right panel). In the LL/2 and B16-F10 tumor models, although radiotherapy with 15 Gy or 3 × 5 Gy was effective at controlling the growth of the irradiated tumors, both regimens failed to trigger abscopal effects in the non-irradiated tumors (**Figures 2D, E** and **Supplementary Figures 2C, D**). Radiation did not lead to prolonged mouse survival in the LL/2 and B16-F10 tumor models (**Figures 2D, E**, right panel).

### Abscopal Effect of Radiotherapy Was Dependent on CD8 T Cell Activation and Infiltration Into Non-irradiated Tumors

**Figure 3:**
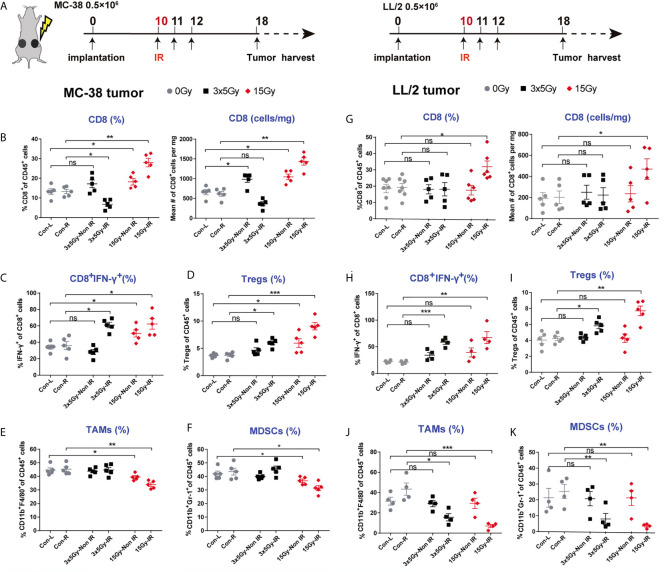
Alteration of the tumor immune microenvironment induced by radiation in highly and poorly immunogenic tumors. **(A)** Tumor-infiltrating immune cells from MC-38 and LL/2 tumors that received 3 × 5 Gy, 15 Gy or sham radiation were analysed 8 days after radiation by flow cytometry. **(B)** Percentages and absolute numbers of CD8 T cells (gating CD45^+^ immune cells) are presented for the MC-38-irradiated and non-irradiated tumors. **(C)** Tumor-infiltrating T cells were stimulated with PMA and ionomycin *in vitro*, and then the percentages of CD8^+^IFN-γ^+^ cells were detected in irradiated and non-irradiated MC-38 tumors. **(D–F)** Percentages of Tregs, MDSCs and TAMs in the irradiated and non-irradiated MC-38 tumors. **(G)** Percentages and absolute numbers of CD8 T cells in the LL/2-irradiated and non-irradiated tumors. **(H)** Percentages of CD8^+^IFN-γ^+^ cells in the LL/2 tumors. **(I–K)** Percentages of Tregs, MDSCs and TAMs in the LL/2-irradiated and non-irradiated tumors. IR, irradiated sides; Non IR, contralateral non-irradiated sides. Representative results from one of at least two independent experiments are shown. *p < 0.05, **p < 0.01, ***p < 0.001, ns, no significance.

The abscopal effect induced by a single high dose of radiation in highly immunogenic tumors prompted us to investigate alterations in the tumor immune microenvironment after radiation (**Figure 3A**). We observed that the percentages and absolute numbers of CD8 T cells in the irradiated and non-irradiated tumors were higher in the 15 Gy radiation group than the control group (**Figure 3B** and **Supplementary Figure 3A**). Correspondingly, CD8 T cells showed more intense IFN-γ production in the irradiated and non-irradiated tumors that received 15 Gy radiation (**Figure 3C** and **Supplementary Figure 3C**). Meanwhile, the percentages of tumor-associated macrophages (TAMs) and myeloid-derived suppressor cells (MDSCs) were reduced in irradiated and non-irradiated tumors after 15 Gy radiation (**Figures 3E, F**). Unexpectedly, the percentages of regulatory T cells (Tregs) were increased in irradiated and non-irradiated tumors of mice receiving 15 Gy or 3 × 5 Gy radiation (**Figure 3D**). In addition, the percentages of CD8 T cells, CD4 T cells and Tregs were all increased in the MC-38-irradiated and non-irradiated tumor-draining lymph nodes (TDLNs) (**Supplementary Figure 4A**). However, for the poorly immunogenic LL/2 tumors, upregulation of the absolute numbers of CD8 T cells, increased the percentages of CD8^+^IFN-γ^+^ T cells and downregulation of immune cells (MDSCs and TAMs cells) was only observed in the irradiated tumors receiving 15 Gy radiation. No significant change in these immune cells was detected in the non-irradiated tumors (**Figures 3G–K** and **Supplementary Figures 3B, D**). Similar results were obtained in the B16-F10 tumor model (**Supplementary Figure 4C**). Moreover, the percentages of CD8 T and CD4 T cells were also increased in the TDLNs of the irradiated tumors but not in the TDLNs of the non-irradiated tumors (**Supplementary Figures 4A**, **B**). Furthermore, to elucidate the role of effector T cells in the abscopal effect of local radiation therapy, we depleted CD8 T cells *via* the systemic administration of an anti-CD8 antibody in MC-38 tumor models. The data showed that depletion of CD8 T cells completely abolished the distant antitumor effect induced by 15 Gy radiation, which was demonstrated by tumor growth (**Supplementary Figure 5**).

### Single High Dose Radiation Reshaped Immune Microenvironment of Non-Irradiated Tumors in Highly Immunogenic Tumors

**Figure 4:**
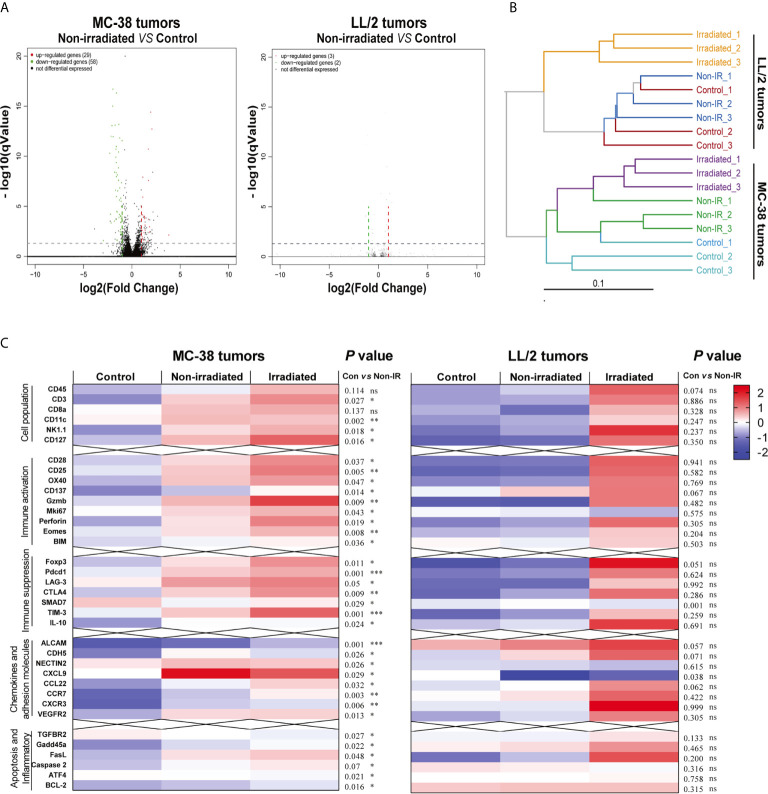
Differentially expressed genes of irradiated tumors, non-irradiated tumors and sham-radiated tumors in the MC-38 and LL/2 tumors. **(A)** Volcano plots for gene expression of non-irradiated tumors and sham-radiated tumors in the MC-38 and LL/2 tumor models. The red and green dots represent up- and downregulated DEGs (differentially expressed genes), respectively. **(B)** Cluster tree showing the dissimilarity and relevance within the irradiated tumors, non-irradiated tumors and sham-radiated tumors in the MC-38 and LL/2 tumors. **(C)** Heat map of immune-related gene expression in the MC-38 tumors and LL/2 tumors 8 days after radiation, including markers of immune cell populations and genes involved in immune activation, immune suppression, cell adhesion and inflammation (n = 3). To compare the immune-related gene expression levels between the non-irradiated tumors and sham-radiated tumors, RNA-seq data were normalised and analysed by the Mann–Whitney test. *p < 0.05, **p < 0.01,***p < 0.001, ns, no significance.

To further explore the immunomodulatory mechanisms of radiation in highly or poorly immunogenic tumors, we harvested the irradiated tumors, non-irradiated tumors and control tumors of MC-38 and LL/2 cells and performed mRNA sequencing (RNA-seq) on day 8 after irradiation. Volcano plots were used to visualise differential immune gene expression between the non-irradiated tumors and control tumors. The results showed that 29 and 58 immune genes were upregulated and downregulated in the MC-38 tumors, respectively, while only three and two genes were upregulated and downregulated in the LL/2 tumors, respectively (**Figure 4A**). The hierarchical cluster analysis showed that the dissimilarity of immune gene expression between the non-irradiated tumors and control tumors was most similar in the LL/2 model while a high-dimensional space of gene expression was found in the MC-38 model (**Figure 4B**). Heat maps were utilised to exhibit the expression of immune-related genes, including markers of immune cell populations, immune activation, immune suppression and the tumor microenvironment. We found that the non-irradiated tumors displayed upregulated immune gene expression compared with the control tumors in the MC-38 model. In contrast, significant differences in gene expression between the non-irradiated tumors and control tumors were not observed in the LL/2 model (**Figure 4C**).

**Figure 5:**
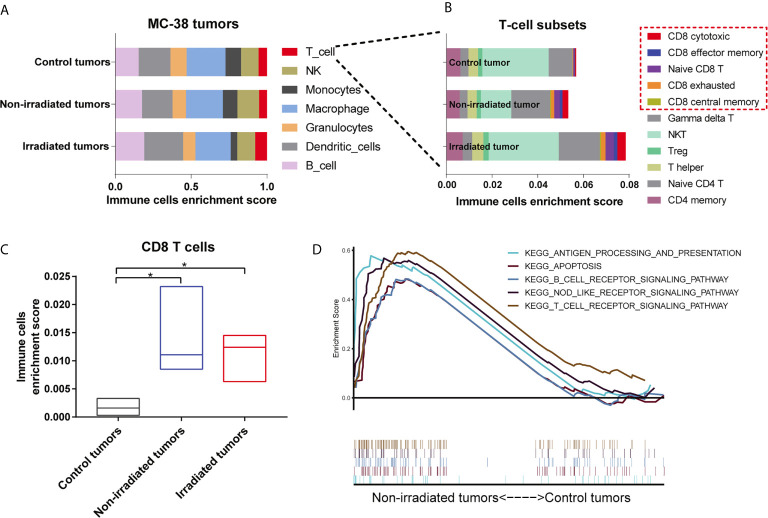
Single high-dose radiation triggered the immune response of non-irradiated tumors in the MC38 model. **(A, B)** Distributions of 7 immune cell and 11 T cell subsets in the irradiated tumors, non-irradiated tumors and control tumors of the MC38 model were assessed by immune cell abundance identifier (immuCellAI-mouse). **(C)** Enrichment scores of CD8 T cells were significantly higher in both irradiated and non-irradiated tumors than in control tumors. **(D)** Significantly enriched KEGG pathways from the GSEA in non-irradiated tumors. *p < 0.05.

Next, an online tool named ImmuCellAI-mouse was used to estimate the abundance of seven immune cells, including 11 T-cell subsets, based on the gene expression profile from the RNA-seq data (9). Our results demonstrated that macrophages and dendritic cells accounted for the majority of immune cell subsets in the MC-38 tumors and T cells were significantly abundant in both the irradiated and non-irradiated tumors compared with the control tumors (**Figure 5A**). Among these T-cell subsets, CD8 T cells increased significantly in both the irradiated and non-irradiated tumors (**Figure 5C** and **Supplementary Figure 6**), including CD8 cytotoxic cells, CD8 central memory cells, CD8 effector memory cells, CD8 exhausted cells and naive CD8 T cells (**Figure 5B**). In addition, a GSEA of signalling pathway enrichment in the non-irradiated tumors compared to the control tumors in the MC-38 model was performed. According to the KEGG enrichment results, pathways related to immune responses, including the T cell receptor signalling pathway, NOD-like receptor signalling pathway, antigen processing and presentation pathway, were upregulated in the non-irradiated tumors (**Figure 5D**).

### Tumor Immunogenic Subtypes Were Associated With the Radiological Response and Clinical Outcome of Patients Receiving Radiotherapy

**Figure 6:**
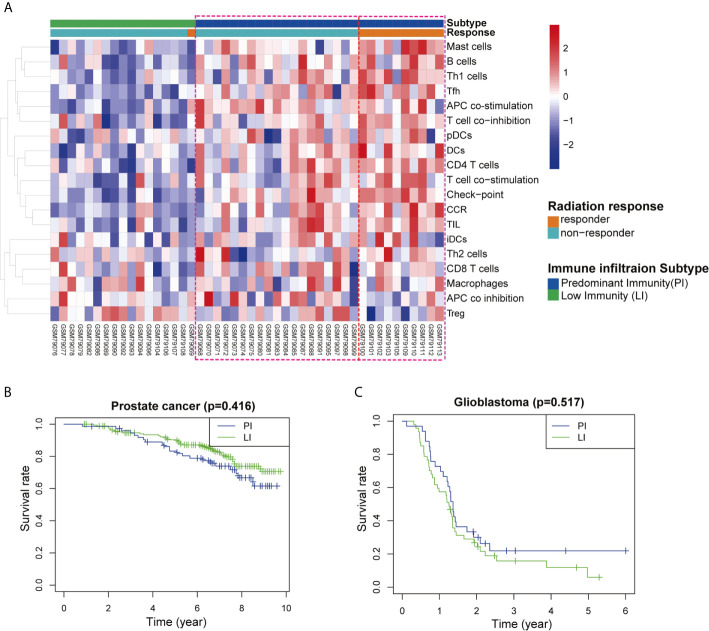
Association between immune profiles of the tumor microenvironment and radiological response in three types of cancer patients. **(A)** Unsupervised clustering of 46 rectal cancer patients who received radiotherapy. The tumor microenvironment was divided into predominant immune groups (PI) and low immune (LI) groups according to single-sample gene-set enrichment analysis (ssGSEA) scores of 29 immune cell types. Patients were classified into two types of radiation response (responder and nonresponder) based on a semiquantitative classification system. **(B, C)** Kaplan–Meier survival curves for the PI and LI groups of cancer patients with **(B)** prostate cancer and **(C)** glioblastoma.

Tumor immunogenicity varies considerably among different types of cancer, and tumor mutation burden (TMB) was recently proven to be positively associated with the immunogenicity of a variety of tumors. Previous studies have shown that rectal cancer is a highly immunogenic tumor with high TMB and prostate cancer and glioblastoma are low immunogenic tumors with low TMB (12–14). Here, we utilised GEO databases to explore the association of radiotherapeutic effects and immune infiltration between the low TMB and high TMB tumors. A total of 29 immune-related gene sets linked to immune infiltration were applied to characterise the two major immunogenicity subtypes in these types of cancer, namely, the predominant immune subtype (PI) and low immune subtype (LI). In the rectal cancer database (GSE35452), patients were classified as “responders” when tumors were assigned a regression grade of 2 or 3 and as “nonresponders” when tumors were assigned a regression grade of 0 or 1 (15). Forty-six tumor samples were divided into the PI group (17/46) and LI group (29/46) according to the ssGSEA scores. Our analysis clearly showed that most radiotherapy responders belonged to the PI group (**Figure 6A**), suggesting a good prognosis among high immune infiltration patients. However, differences in survival were not observed between the PI group and the LI group of prostate cancer (GSE116918) and glioblastoma (GSE7696) (**Figures 6B, C**).

## Discussion

Abscopal effects are rare phenomena in which tumors outside of the irradiated field regress due to the systemic antitumor effects of local radiotherapy (2). Previous reports documented that the majority of clinical cases of radiation-induced abscopal effects occurred in immunogenic tumors, such as renal cell carcinoma, melanoma and hepatocellular carcinoma (16–18), indicating that the abscopal effect was correlated with tumor immunogenicity. Tumor immunogenicity differs in different types of advanced solid tumors. In this study, we stratified tumors as highly and poorly immunogenic according to the densities of intratumoral immune cell infiltration and the MHC-I expression of tumor cells. We found that the abscopal effect was only induced in the highly immunogenic tumor models (MC-38 and E.G7-OVA) but not in the poorly immunogenic tumor models (LL/2 and B16). Many studies have revealed that irradiation stimulates the immune system through numerous pathways, including the release of previously hidden tumor-associated antigens (TAAs) and immune-stimulatory molecules from tumors, which could activate and prime an antitumor immune response (19–21). Considering that highly immunogenic tumors were more likely to harbour neoantigens and an associated increase in cytotoxic T cells occurred in the tumor microenvironment, it would seem reasonable to speculate that the abscopal effect of radiation may be correlated with tumor immunogenicity.

Apart from the tumor microenvironment, the dosage and fraction regimen of radiation may also have a substantial impact on the abscopal antitumor effect. To address this question, two different treatment regimens, namely, a single dose of 15 Gy or fractionated dose of 3 × 5 Gy irradiation, were applied in this study. Our results demonstrated that single high-dose radiation (15 Gy) was optimal for eliciting a robust local immune response and inducing an abscopal effect in highly immunogenic tumors compared to 3 × 5 Gy radiation and radiotherapy was insufficient to elicit an abscopal effect in poorly immunogenic tumors. Thus far, an “optimal” radiation scheme has not been developed for the induction of abscopal effects. Dewan et al. showed that the abscopal effect was only induced in fractionated regimens (22). Conversely, other groups reported that the antitumor immunity elicited by high single-dose radiation was more potent than that by fractionated treatments (23). However, most of these previous studies applied single high-dose radiation or hypofractionated regimens (≥6 Gy) to induce abscopal effects. In our opinion, these paradoxical results are based on differences in immune-relevant changes in the tumor microenvironment induced by distinct radiotherapy regimens.

Many mechanisms have been proposed to elucidate the abscopal effect of radiation (24). Previous studies have revealed that local irradiation can activate a cascade of innate and adaptive immunity through numerous pathways, including the release of DAMPs, activation of the STING (stimulator of interferon genes) signalling pathway, cross-presentation of TAAs, etc. (25–27). Our latest research study confirmed that radiation-induced local immune responses are largely dependent on CD8 T cells, which is consistent with other reports (28). Considering that tumor immunogenicity was correlated with a higher level of immune cell infiltration (**Figures 1A–C** and **Supplementary Figures 1B, C**), immune cell infiltration in tumors should play a crucial role in mediating the response to radiotherapy. In this study, we found that CD8 T-cell infiltration and IFN-γ production were upregulated by 15 Gy radiation in both the irradiated and non-irradiated tumors with high immunogenicity, which was accompanied by the downregulation of suppressive immune cells (MDSCs and TAMs) at the tumor sites (**Figure 3** and **Supplementary Figure 3**). However, the infiltration of CD8 T cells was decreased in the irradiated tumors receiving 3 × 5 Gy radiation (**Figure 3B**). These results have several potential explanations, including fractionated radiation-induced effector CD8 cell death, which dampens the antitumor immune response (29), and the spatiotemporal dynamics of CD8 T cell infiltration after radiation. Radiation-induced CD8 T cell infiltration occurred in a narrow time window (30). Furthermore, cytotoxic T cells displayed a radiation-sensitive phenotype that might be affected during reirradiation (31). Interestingly, although CD8 T cells were decreased in the irradiated tumors of the 3 × 5 Gy group, abscopal effects were still observed in the 3 × 5 Gy group (**Figure 2B** and **Supplementary Figure 2A**). These results confirmed that fractionated irradiation was directly toxic to the T cells in the irradiated tumors. Compared with the irradiated tumors, higher concentrations of CD8 T cells were observed in the non-irradiated tumors after radiation (3 × 5 Gy and 15 Gy) (**Figure 3B** left), indicating that a systemic antitumor immune response was triggered by the newly infiltrated CD8 T cells after radiation treatment. In addition, upregulation of Treg infiltration induced by radiation (3 × 5 Gy and 15 Gy) was observed in the irradiated and non-irradiated tumors (**Figure 3D**). Treg cells are an important regulator of inflammation and homeostasis of the immune system (32). An increase in Treg cells has been widely reported as a mechanism underlying the radiation resistance and immunoregulatory function of irradiated tumors, and it is preceded by the infiltration of CD8 T cells. However, the decrease in CD8 T cells and increase in Tregs observed in the irradiated tumors that received 3 × 5 Gy may have been related to the decrease in CD8 T cells after fractionated irradiation and the presence of radioresistant Treg cells inside the tumors. Moreover, radiation can cause the release of immunosuppressive cytokines (TGF-β and IL-10) and increase the fraction of Tregs in the spleen and circulation (33). Therefore, one explanation for the increase in Tregs in non-irradiated tumors was that radiation enhanced the recruitment of circulating Tregs to abscopal tumors. Taken together, these results indicated that high-dose radiation alone increased CD8 T cell infiltration and reduced the percentage of MDSCs and TAMs in non-irradiated tumors, which induced an abscopal effect in highly immunogenic tumors. Some barriers in the tumor microenvironment might prevent the responding immune cells from migrating and infiltrating into non-irradiated tumor sites of poorly immunogenic tumors.

Our results revealed that local irradiation induced a strong systemic immune response and altered the gene expression profiles of non-irradiated tumors with high immunogenicity and showed that irradiation alone was not sufficient to change non-irradiated tumors with poor immunogenicity. Intriguingly, the increase in CD8 T-cell infiltration and upregulation of immune-related genes were observed in the irradiated tumors but not in the non-irradiated tumors with poor immunogenicity. Collectively, these immune characterisations could at least partially explain why radiation alone could trigger abscopal effects in highly immunogenic tumors but not in poorly immunogenic tumors. Our findings indicated that tumor-mediated tolerance or barriers could be overcome by radiation-induced systemic antitumor responses in highly immunogenic tumors and primed CD8 T cells could recognise and attack both local tumors and distant tumors outside the radiation field. In contrast, low immunogenicity indicated that TAAs were downregulated in tumors and evasion of host immunity occurred in the tumor microenvironment, which lacked chemokine-mediated trafficking and showed poor adaptive immune cell activation (8, 11). The rarity of the abscopal effect suggests that even primed antitumor CD8 T cells could not overcome a suppressive tumor microenvironment with low infiltration of responding immune cells. Radiation has been reported to modulate tumor immunogenicity in various tumor types by converting the biology of surviving tumor cells to render them more sensitive to T cell-mediated immunity (34, 35). Here, our data further confirmed that the immune profiles of the tumor microenvironment played a critical role in whether an abscopal effect occurred (36).

In clinical studies, high TMB represents genomic instability and enriched tumor neoantigens, which is associated with increased tumor immunogenicity. Recent evidence suggested that TMB and immune cell infiltration were promising biomarker for immunotherapy response in cancer patients (37–41). Valero et al. reported that combining neutrophil-to-lymphocyte ratio (NLR) with TMB provided more accurate prediction for immunotherapy. They found that NLR-low/TMB-high group had higher immunotherapy response rates and better outcome (40). The results of these studies raised the intriguing possibility that tumor immunogenicity combined with immune infiltration may be used as predictive biomarkers in the context of radiotherapy. Our analysis of data from the GEO database suggested that there was a positive correlation between immune infiltration and radiotherapy effects in high immunogenicity tumors. Tumor-infiltrating immune cells represent actual conditions of the tumor immune microenvironment. Radiotherapy promotes the release of tumor neoantigens in highly immunogenic tumors, which is beneficial to activate the immune cells and be able to recognize and attack cancer cells. In addition, tumor microenvironment and immune status are associated with peripheral blood immune status. Immune status in peripheral blood provided a comprehensive view of the status of the immune system and correlated with T cell function in the tumor microenvironment (42). Many studies have revealed that peripheral blood immune cell subsets can be served as biomarkers to predict immunotherapy efficacy (43). Zhou et al. reported that peripheral blood immune cells including NKT cells and neutrophils can be used as predictive biomarkers for immunotherapy (44). Of note, peripheral blood can be obtained easily and be repeated compared to tissue biopsy, particularly during the evolving phases of therapy. However, relationship between tumor immunogenicity and peripheral blood immune status remains unknown. Future considerations on the role of local/systemic immune status and tumor immunogenicity in radiotherapy should be explored.

Altogether, our findings indicated that tumor immunogenicity is a critical determinant of the abscopal effect of cancer radiotherapy and showed that the systemic antitumor response generated by radiation may be based on differences in the immune infiltration densities and immune activities between highly immunogenic tumors and poorly immunogenic tumors. Furthermore, in our study, PD-1 and TIM-3 expression was increased in CD8 cells after radiation, thereby representing the exhausted phenotype by failure to produce IL-2 and IFN-γ (45). The upregulation of PD-L1 has also been observed in irradiated tumors in many reports (46, 47). This evidence provides an opportunity for PD-1/PD-L1 blockade to normalise host immunity against tumors. Based on these data, follow-up studies were carried out to examine whether the combination of radiotherapy and PD-1 blockade could induce different antitumor immunity and abscopal effects between highly immunogenic and poorly immunogenic tumors.

## Conclusion

In summary, our study suggested a direct connection between the abscopal effect and tumor immunogenicity. Although local radiation has the ability to convert the irradiated tumor into an immunogenic hub, it fails to induce an abscopal effect in poorly immunogenic tumors. However, in highly immunogenic tumors, single high-dose radiation was optimal for eliciting robust CD8 T-cell infiltration and inducing an abscopal effect. These findings provide valuable information to improve our understanding of the abscopal effect and boost the application of radiotherapy for the treatment of both local and metastatic disease in the clinic.

## Data Availability Statement

The original contributions presented in the study are publicly available. This data can be found here: https://doi.org/10.6084/m9.figshare.14414579.

## Ethics Statement

The animal study was reviewed and approved by the Fujian Medical University Institutional Animal Care and Use Committee.

## Author Contributions

J-ZL and Q-YZ conceived and designed the study. J-ZL and YL performed the experiments. Y-YZ and LH cooperated in the establishment of tumor models. L-LZ and LC provided technical guidance. J-ZL and LC analyzed the data and drafted the manuscript. Q-YZ supervised the study and reviewed the manuscript. All authors contributed to the article and approved the submitted version.

## Funding

This study was supported by National Natural Science Foundation of China (No. 82073350), Fujian Provincial Committee of Natural Science and Technology (No. 2020J02039), and Education and Scientific Research Foundation for Young and Middle-aged teachers in Fujian Province (No. JAT160210).

## Conflict of Interest

The authors declare that the research was conducted in the absence of any commercial or financial relationships that could be construed as a potential conflict of interest.
