# Supplementary material for: Abscopal Effects of Local Radiotherapy Are Dependent on Tumor Immunogenicity
Source: Front Oncol. 2021 Jun 24;11:690188. doi: 10.3389/fonc.2021.690188 (PMC8264447; doi:10.3389/fonc.2021.690188)
Supplement: Supplementary file 1 [file Presentation_1.pdf]

Supplementary Material

Supplementary Figures

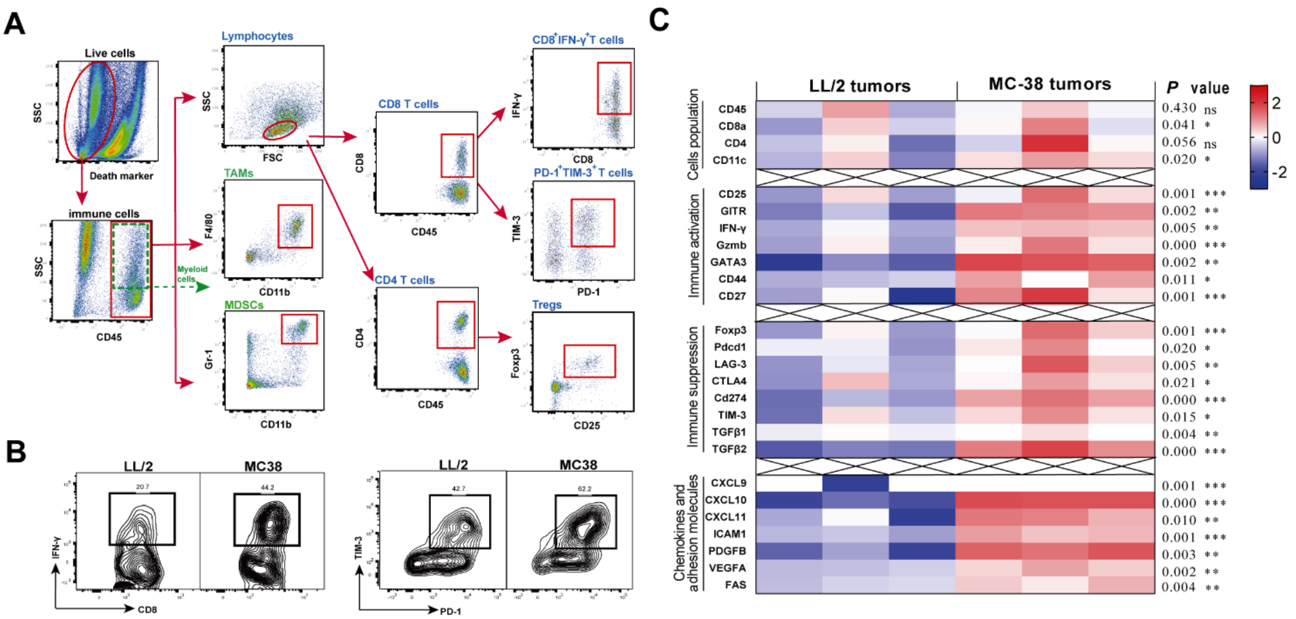

Supplementary figure 1. The percentage of infiltrating CD8 T cells and the expression level of immune-related genes was higher in MC-38 tumors than that in LL/2 tumors. **(A)** Gating strategy for flow-cytometry analysis of the live cells, CD45$^+$ cells, Lymphocytes, CD4$^+$ T cells, CD8$^+$ T cells, MDSCs, TAMs, Tregs, CD8$^+$IFN-γ$^+$ T cells and PD-1$^+$TIM-3$^+$ T cells. **(B)** Representative plots of percentages of CD8$^+$IFN-γ$^+$ T cells and PD-1$^+$TIM-3$^+$ (gating CD45$^+$ CD8$^+$ cells) which were analyzed by flow cytometry. **(C)** Heatmaps of differential immune-related gene between MC-38 tumors and LL/2 tumors, including markers of immune cell populations and genes involved in immune activation, immune suppression, cell adhesion and inflammation (n=3).

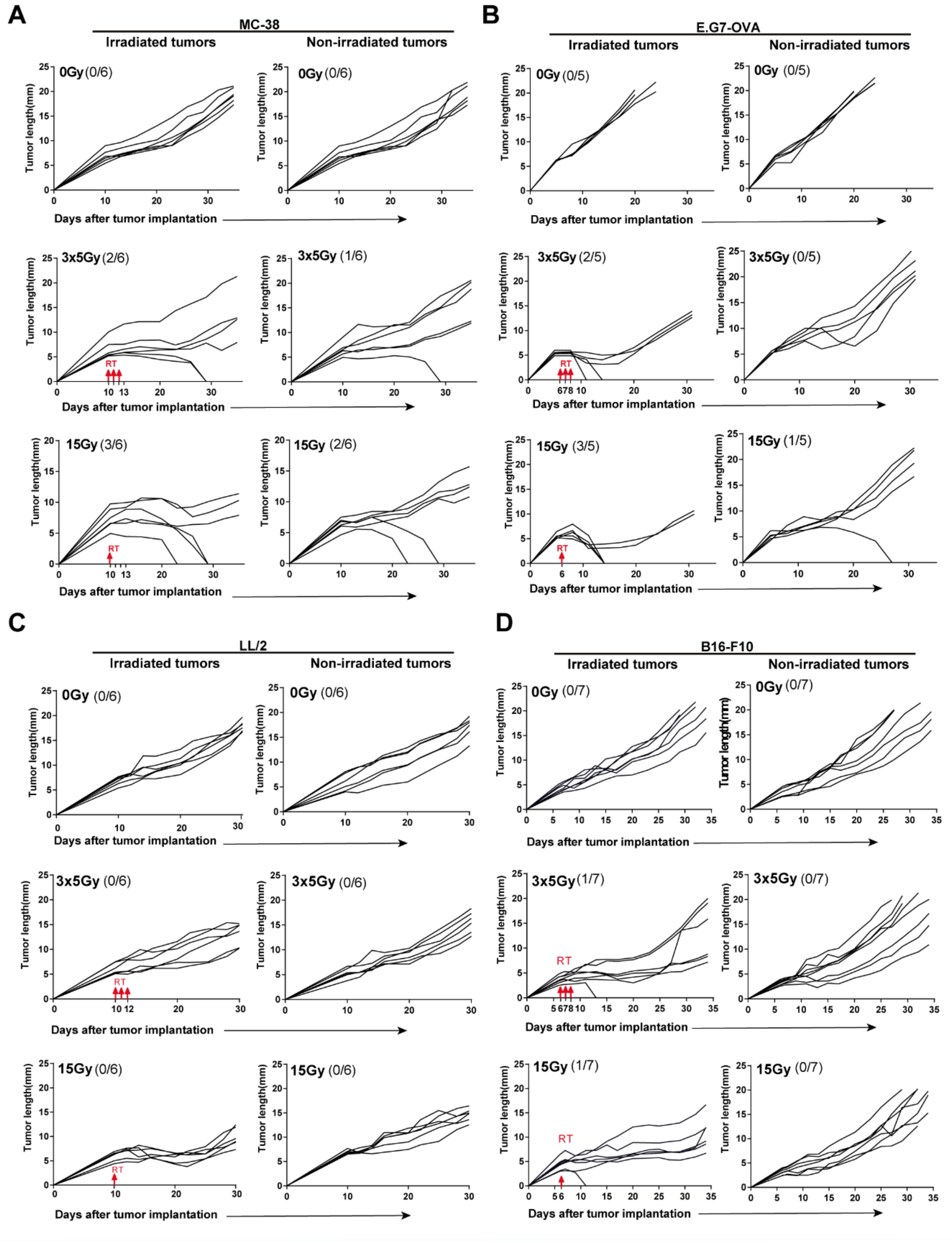

Supplementary figure 2. Individual growth of irradiated and non-irradiated tumors in mice bearing **(A)** MC-38 tumors (n=6), **(B)** E.G7-OVA tumors (n=5), **(C)** LL/2 tumors (n=6) and **(D)** B16-F10 tumors (n=7).

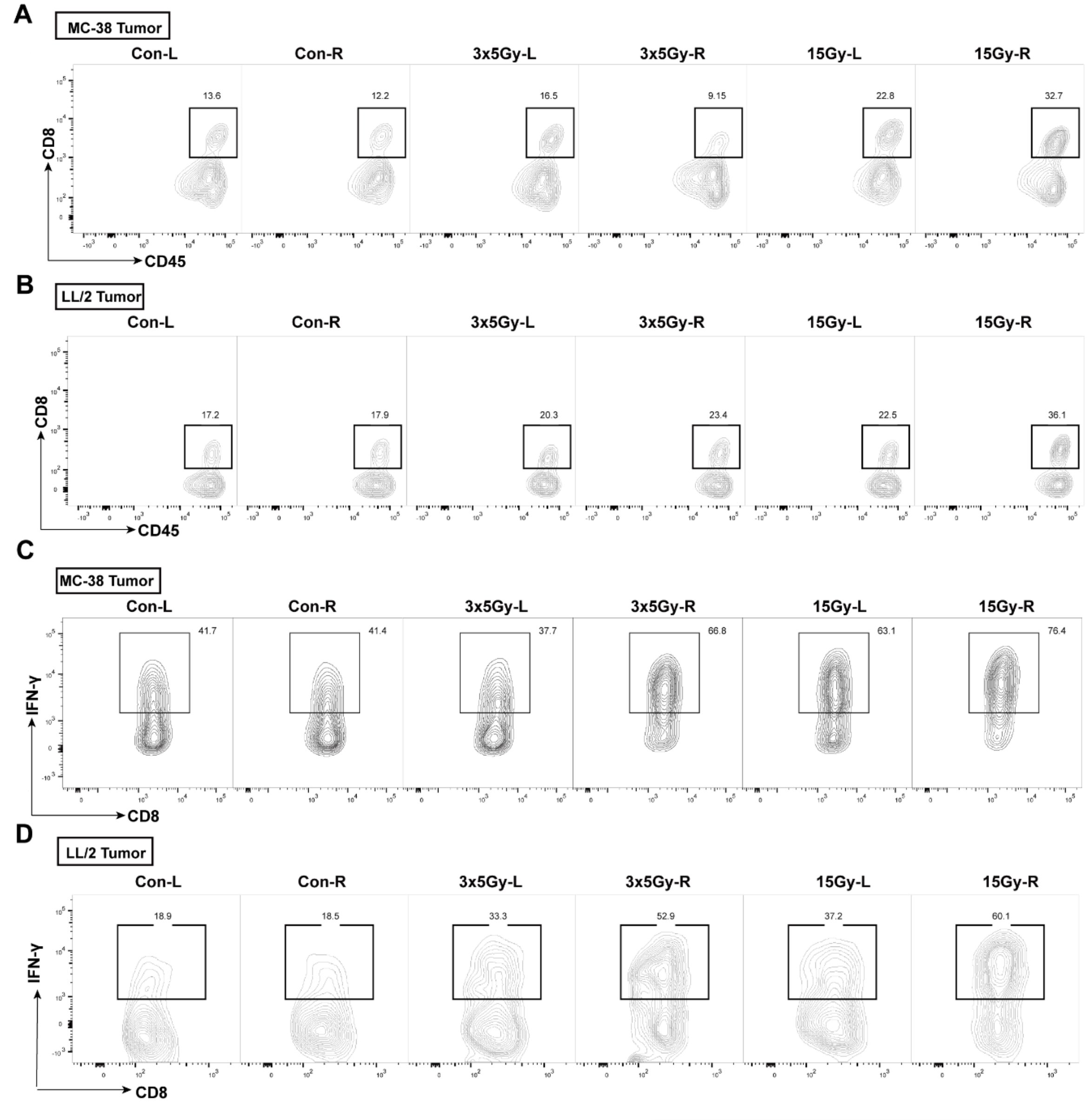

Supplementary figure 3.| The percentages of CD8 T cells (gating CD45$^+$ cells) in the tumor microenvironment of MC38 tumors and LL/2 tumors. **(A-B)** Representative plots of percentages of CD8 T cells were presented in irradiated and non-irradiated tumors of MC38 tumor model **(A) and** LL/2 tumor model **(B).** Representative plots of percentages of CD8$^+$IFN-γ^+ T cells (gating CD3$^+$ CD8$^+$ cells) in irradiated and non-irradiated tumors of MC38 tumor model **(C) and** LL/2 tumor model **(D)**. * $p<0.05$, ** $p<0.01$, *** $p<0.001$.

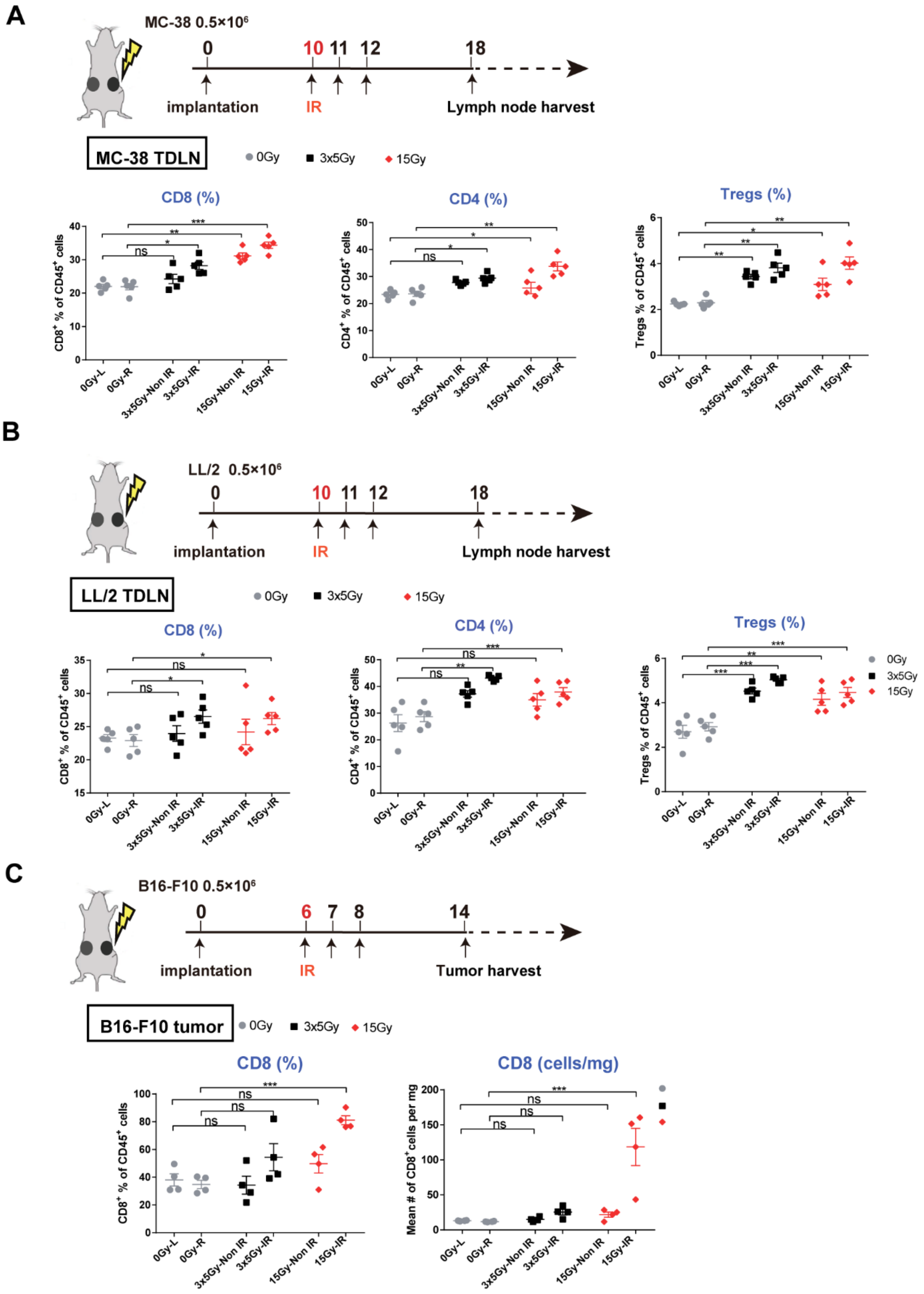

Supplementary figure 4. The percentages of T cells subsets in tumor draining lymph nodes (TDLNs) and B16-F10 tumors. **(A-B)** Percentages of CD8 T cells, CD4 T cells and Tregs in TDLNs derived from **(A)** MC-38 models and **(B)** LL/2 models. **(C)**Percentages and absolute numbers of CD8 T cells were presented in irradiated and non-irradiated B16-F10 tumors. IR: irradiated sides; Non IR:

contralateral non-irradiated sides. Representative results from one of at least two independent experiments were shown. * $p<0.05$, ** $p<0.01$, *** $p<0.001$.

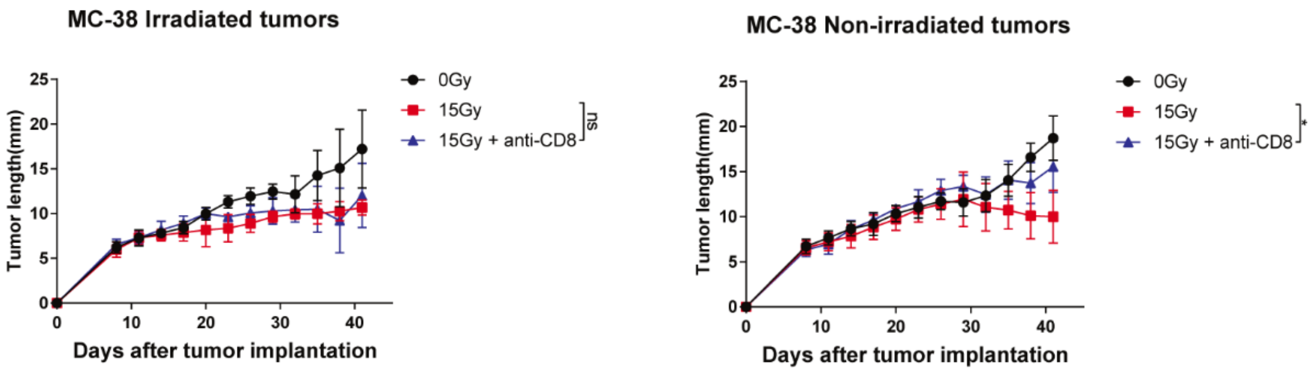

Supplementary figure 5. Depletion of CD8 T cells in MC-38 tumors. Starting from the day before 15Gy radiation, 200 µg CD8 depleting antibody (clone 53.6.7) was intra-peritoneal injected every 4 days in mice bearing MC-38 tumors.

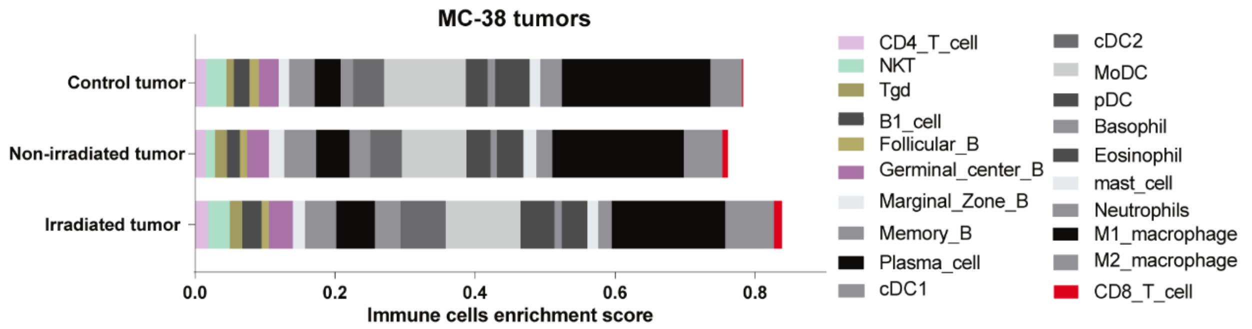

Supplementary figure 6. Distributions of 20 immune cells among irradiated tumors, non-irradiated tumors and control tumors in MC-38 model were estimated using the immuCellAI-mouse tool.
